# Draft Genome Sequence of Halomonas titanicae Strain TAT1, a Hydrocarbon-Oxidizing Halophilic Bacterium Isolated from a Petroleum Reservoir in Russia

**DOI:** 10.1128/MRA.01255-20

**Published:** 2020-11-25

**Authors:** Denis S. Grouzdev, Diyana S. Sokolova, Andrey B. Poltaraus, Tamara N. Nazina

**Affiliations:** a SciBear LLC, Tallinn, Estonia; b Winogradsky Institute of Microbiology, Research Center of Biotechnology, Russian Academy of Sciences, Moscow, Russian Federation; c Engelhardt Institute of Molecular Biology, Russian Academy of Sciences, Moscow, Russian Federation; University of Delaware

## Abstract

The draft genome sequence of a moderately halophilic bacterium, Halomonas titanicae strain TAT1, isolated from production water of the Romashkinskoe oilfield (Russia) is presented. The genome is annotated for elucidation of the metabolic pathways involved in hydrocarbon degradation and nitrate reduction in petroleum-contaminated hypersaline environments.

## ANNOUNCEMENT

Aerobic halophilic oil-oxidizing bacteria are promising for application in biotechnologies for enhanced oil recovery and for bioremediation of oil-contaminated hypersaline soil and marine environments (1, 2). Halomonas titanicae strain TAT1 (VKM B-3500D) was isolated from highly mineralized production water of the Romashkinskoe oilfield (Russia) (54°28′41″N, 52°06′27″E) (3). The strain TAT1 was capable of growth under high salinity (optimum, 5 to 10% NaCl [wt/vol]) both aerobically on mineral medium with crude oil (with biosurfactant production) and anaerobically on complex media with sugars and proteinaceous substrates, reducing nitrate to dinitrogen (4). The goal of the present work was to sequence the TAT1 genome in order to obtain more insight into specific traits related to hydrocarbon oxidation and nitrate reduction at high salinity.

The strain was grown aerobically at 25 to 30°C in LB medium with 5% NaCl (wt/vol), and cells were harvested after 7 days of cultivation. DNA was purified from the cell biomass using the cetyltrimethylammonium bromide (CTAB) method (5). DNA libraries were prepared with the NEBNext DNA library prep reagent kit for Illumina (New England Biolabs). Next-generation shotgun sequencing of genomic DNA was carried out using the HiSeq 2500 platform (Illumina, Inc., USA) with 150-bp paired-end reads. Raw sequence reads were quality-checked with FastQC v. 11.7 (https://www.bioinformatics.babraham.ac.uk/projects/fastqc/), and low-quality reads were trimmed using Trimmomatic v. 0.36 (6) with the default settings for paired-end reads. Subsequently, the quality-filtered reads were *de novo* assembled with SPAdes v. 3.13.0 using the default settings (7). The estimated completeness and contamination evaluated using CheckM v. 1.0.18 were 100.0% and 0.9%, respectively (8). The taxonomic position of the assembled genome was determined using GTDB-Tk v. 1.0.2 (9). Average nucleotide identity (ANI) and digital DNA-DNA hybridization (dDDH) of genomes were determined using FastANI v. 1.3 (10) and the Genome-to-Genome Distance Calculator (GGDC) v. 2.1 (11), respectively. Identification of protein-coding sequences and primary annotation were performed using the NCBI Prokaryotic Genome Annotation Pipeline (PGAP) (12).

The final assembled 5,303,463-bp-long TAT1 genome sequence comprised 51 scaffolds, with an *N*_50_ value of 381,158 bp, G+C content of 54.6%, and coverage of 285×. The genome sequence contained 4,910 genes, 4,757 of which were protein-coding genes, and 67 coded RNAs. The ANI and dDDH values with the closely related genome of Halomonas titanicae BH1^T^ were 99.8% and 99.2%, respectively, which supported the identification of the strain as a member of this species (13). The TAT1 genome harbors large numbers of genes coding for the degradation of benzoate, biphenyl, gentisate, and *N*-heterocyclic aromatic compounds, assimilatory sulfate reduction, dissimilatory nitrate reduction, and denitrification. This information will be useful for revealing the mechanism of hydrocarbon degradation mediated by *H. titanicae* TAT1 and its potential for bioremediation of petroleum- or nitrate-contaminated hypersaline environments.

### Data availability.

This whole-genome shotgun project has been deposited in DDBJ/ENA/GenBank under the BioProject accession number PRJNA637646. The raw reads were deposited in the Sequence Read Archive (SRA) under accession number SRR11977805. The genomic version described in this paper is version JABWTB010000000.
